# MR diffusion-weighted imaging-based subcutaneous tumour volumetry in a xenografted nude mouse model using 3D Slicer: an accurate and repeatable method

**DOI:** 10.1038/srep15653

**Published:** 2015-10-22

**Authors:** Zelan Ma, Xin Chen, Yanqi Huang, Lan He, Cuishan Liang, Changhong Liang, Zaiyi Liu

**Affiliations:** 1 Department of Radiology, Guangdong General Hospital, Guangdong Academy of Medical Sciences, Guangzhou, Guangdong, 510080, China.; 2 Graduate College, Southern Medical University, Guangzhou, Guangdong, 510515, China.; 3 Department of Radiology, The Affiliated Guangzhou First Hospital, Guangzhou Medical University, Guangzhou, Guangdong, 510180, China.; 4 School of Medicine, South China University of Technology, Guangzhou, Guangdong, 510006, China.

## Abstract

Accurate and repeatable measurement of the gross tumour volume(GTV) of subcutaneous xenografts is crucial in the evaluation of anti-tumour therapy. Formula and image-based manual segmentation methods are commonly used for GTV measurement but are hindered by low accuracy and reproducibility. 3D Slicer is open-source software that provides semiautomatic segmentation for GTV measurements. In our study, subcutaneous GTVs from nude mouse xenografts were measured by semiautomatic segmentation with 3D Slicer based on morphological magnetic resonance imaging(mMRI) or diffusion-weighted imaging(DWI)(b = 0,20,800 s/mm^2^) . These GTVs were then compared with those obtained via the formula and image-based manual segmentation methods with ITK software using the true tumour volume as the standard reference. The effects of tumour size and shape on GTVs measurements were also investigated. Our results showed that, when compared with the true tumour volume, segmentation for DWI(P = 0.060–0.671) resulted in better accuracy than that mMRI(P < 0.001) and the formula method(P < 0.001). Furthermore, semiautomatic segmentation for DWI(intraclass correlation coefficient, ICC = 0.9999) resulted in higher reliability than manual segmentation(ICC = 0.9996–0.9998). Tumour size and shape had no effects on GTV measurement across all methods. Therefore, DWI-based semiautomatic segmentation, which is accurate and reproducible and also provides biological information, is the optimal GTV measurement method in the assessment of anti-tumour treatments.

The subcutaneous xenograft tumour model in nude mice is an ideal model for monitoring responses to anti-tumour treatments, and the gross tumour volume (GTV) is an important index for therapeutic evaluation, especially in radiotherapy treatment planning (RTP)^1^,^2^,^3^. Therefore, non-invasive, accurate and repeatable GTV measurement is critical to evaluate the response to treatment in longitudinal anti-tumour studies^4^. The widely applied method for GTV measurement in subcutaneous mouse xenografts is usually a modified ellipsoidal formula of V = 1/2(AB^2^), where A is the greatest longitudinal diameter and B is the greatest transverse diameter measured with an external calliper^5^. However, this technique for volume measurement demonstrates increased error when measuring irregularly shaped and large hematomas^6^,^7^,^8^. Recently, tumour delineation based on cross-sectional imaging, such as computed tomography (CT) and magnetic resonance imaging (MRI), has shown usefulness in GTV measurement^9^,^10^.

Compared with other imaging modalities, MRI holds great promise in animal tumour studies by providing excellent spatial resolution of parenchymal organs and invaluable functional information about organs and tumours using functional MRI (fMRI) such as dynamic contrast-enhanced imaging (DCE) and diffusion-weighted imaging (DWI) techniques, which are widely accepted and have been used in animal and clinical studies^11^,^12^. Although morphological MRI (mMRI), such as T1-weighted imaging (T1WI) and T2-weighted imaging (T2WI), has been used for GTV measurement with high resolution, it cannot provide any functional information about tissues. On the other hand, DCE MRI can provide valuable functional information but has the disadvantage of requiring the intravenous administration of contrast media^12^. DWI is the only *in vivo* method to probe water diffusion by using the random microscopic motion of water molecules in tissues without the use of any intravenous contrast agent; it can be easily applied in the evaluation of treatment responses^3^. Many studies have previously shown that both T2WI and DWI could be used for GTV measurement; however, their findings were not in concordance. Wolf *et al.* demonstrated that the intra- and inter-observer variability based on DWI was greater than that of T2WI, and the median GTV measured with DWI was higher than that of T2WI^13^. However, Regini *et al*. presented completely different results in their recent study^14^. In addition, diffusion-weighted intravoxel incoherent motion (IVIM) imaging with multiple b values fitted to a bi-exponential model has been widely studied for its capability of characterizing diffusion and perfusion effects in normal and diseased tissues^15^,^16^. To our knowledge, the effects of different b values on GTV measurement have not been reported.

In addition to the controversy over MRI sequences for GTV measurements, the imaging segmentation method is another confounding factor. Typically, tumour volume is calculated via the manual segmentation method in MRI studies^13^,^14^. However, this method is time-consuming and has large inter-observer variability because no recognized criteria exist to delineate tumour boundaries^17^. Hence, automatic tumour segmentations supported by software have been proposed^18^. However, because automatic segmentation often fails to match manual tumour delineation, these methods have not been widely applied in clinical practice^18^. Recently, a semiautomatic segmentation method, the GrowCut algorithm within the freely available software 3D Slicer (Brigham and Women’s Hospital, Boston, MA, USA), was shown to be more accurate and stable than manual delineations by experts in tumour segmentation^19^,^20^,^21^. To the best of our knowledge, there are currently no reports comparing the accuracy and reproducibility of GTV measurements of subcutaneous tumour volumetry using 3D Slicer with DWI versus other measuring methods including image-based manual segmentation and the formula method using the true tumour volume as the reference standard.

Therefore, the aim of this study was to evaluate different methods, including the formula method and the semiautomatic and manual segmentation methods for mMRI or DWI, with respect to their accuracy and reproducibility in GTV measurement in a subcutaneous tumour-xenograft nude mouse model with the true volume of the resected tumour as the reference standard.

## Results

The described volume measurements *in vivo* and *ex vivo* were successfully performed in 35 mice. No significant differences were observed between two repeated measurements taken by the same person using all the above-described methods (P = 0.119–0.853). Therefore, we calculated the mean volume of two measurements for further analysis with each method.

### Comparison of GTV measurements via mMRI- and DWIs-based segmentation and the formula method with the true tumour volume

The mean and standard deviation (SD) for each GTV are summarized in Table 1. The results of the comparison between *in vivo* and *ex vivo* GTV measurement methods are shown in Table 2. The mean GTV obtained with the formula method had the largest measured volume, which was then followed by the volumes obtained with the segmentation software for both 3D Slicer and ITK with mMRI and DWI. The true tumour volumes were the smallest.

As shown in Tables 1 and 2, the GTV calculated by the formula method was 1.99 ± 1.40 cm^3^ and was significantly larger than the true tumour volume (1.72 ± 1.35 cm^3^) (P < 0.001); the GTVs calculated from mMRI-based segmentation by ITK (1.77 ± 1.36 cm^3^) and 3D Slicer (1.77 ± 1.35 cm^3^) were also significantly larger than the true tumour volume (ITK: P < 0.001; 3D Slicer: P < 0.001). Furthermore, when taking tumour size and shape into account, GTVs of subgroups obtained via the formula method and mMRI-based manual and semiautomatic segmentation were all larger than the true tumour volume (Formula: P = 0.000–0.037, ITK: P = 0.000–0.011; 3D Slicer: P = 0.001–0.025).

The GTVs obtained from 3 sets of DWIs (b = 0, 20, 800 s/mm^2^) were 1.72 ± 1.34 cm^3^, 1.73 ± 1.33 cm^3^ and 1.73 ± 1.34 cm^3^, respectively, via the manual segmentation method, and all were 1.73 ± 1.35 cm^3^ with the semiautomatic segmentation method (Table 1). No significant differences were observed between DWI-based segmented GTVs and true tumour volumes (ITK, P = 0.060–0.671; 3D Slicer, P = 0.064–0.351) (Table 2). There were no significant differences in GTVs obtained via DWI-based manual and semiautomatic segmentation as compared to the true tumour volume with regard to tumour size and shape (ITK: P = 0.050–0.972; 3D Slicer: P = 0.171–0.608) (Table 2).

### Comparison of GTV measurements between manual and semiautomatic segmentation methods based on different images

Table 3 shows that there were no significant differences in image-based GTV measurements between the manual (ITK) and semiautomatic segmentation (3D Slicer) methods when applied to either mMRI or DWIs (mMRI, P = 0.232; DWIs, P = 0.087–0.787).

When taking tumour size and shape into account, there were no significant differences in GTV measurements between ITK and 3D Slicer for all image sets (mMRI: P = 0.054–0.877; DWI_b0: P = 0.220–0.257; DWI_b20: P = 0.308–0.740; DWI_b800: P = 0.449–0.721).

### Reproducibility of GTV measurements by the manual and semiautomatic segmentation methods based on DWI

As shown in Tables 1 and 2, GTVs calculated by the formula method, as well as by both segmentation methods applied to mMRI, were significantly different from the true tumour volumes, suggesting that their accuracy was not satisfactory. As accuracy is a critical factor for a suitable method, these methods were not included to evaluate their reproducibility for GTV measurement. In this study, we used the intraclass correlation coefficient (ICC) to assess the intra-observer agreement for the evaluation of reproducibility for the GTV measurement methods.

Table 4 shows that the ICC ranged from 0.9996 to 0.9998 with the manual method (ITK) and reached 0.9999 with the semiautomatic method (3D Slicer) for DWI. All segmentation methods showed excellent intra-observer measurement reproducibility, which was further increased with the semiautomatic method. When dividing the cohort into subgroups based on size and shape, the larger volume group with true tumour volumes equal to or larger than the median size of 1.13 cm^3^ (ICC = 0.9993–0.9999) demonstrated better measurement reproducibility than the smaller volume group (ICC = 0.9908–0.9978); furthermore, the measurement reproducibility of the irregular tumour group (ICC = 0.9996–0.9999) was slightly better than that of the regular tumour group (ICC = 0.9994–0.9998) (Table 4). Of the three different b values (b = 0, 20, 800 s/mm^2^), the b value of 0 s/mm^2^ demonstrated the highest measurement reproducibility (ICC = 0.9998–0.9999) for both manual segmentation and semiautomatic segmentation (Table 4).

## Discussion

GTV measurement is critical for all tumours as it is necessary for high accuracy therapy evaluation^1^. Subcutaneous tumour-xenografted nude mice as a pre-clinical model have been widely used to assess tumour treatment responses. In this study, we measured subcutaneous GTV in a liver cancer xenografted nude mouse model by employing the formula method and MRI-based segmentation methods. We attempted to obtain a non-invasive, accurate and reproducible GTV measurement method for the evaluation of anti-tumour therapies in animal models.

Our results showed that the tumour volumes measured by the modified ellipsoidal formula method were significantly greater than the true tumour volumes. We speculate that the overestimation of GTV may be due to the double layer of skin surrounding the tumour, which was also measured by external callipers and included for volume calculations. Moreover, given that this formula applies to tumours with a regular ellipsoid shape, when applied to cases with irregular shapes it may result in large variability and low reproducibility of GTV measurements^22^. Therefore, subcutaneous GTV measured by the formula method has a certain error range.

Morphological imaging (e.g., CT, MR T1WI or T2WI) and functional imaging (e.g., MR DWI and positive emission tomography, PET) have been used for the delineation of GTV measurements^10^,^13^,^18^,^23^,^24^. Among these imaging modalities, MRI provides the best soft tissue contrast and spatial resolution compared to other modalities^4^. Although the automatic segmentation method requires the least amount of user intervention, it is difficult to achieve satisfactory results when inspected by experienced physicians^17^. Therefore, semiautomatic segmentation appears to be more feasible than automatic segmentation. To our knowledge, we are the first team to study MRI-based semiautomatic segmentation using 3D Slicer by comparing manual segmentation with the true resected tumour volume as the reference standard. Furthermore, in our study, evaluation of MRI-based GTV segmentation was performed based on three sets of IVIM DWIs in which three representative b values out of 12 b values were used for GTV segmentation.

Our study result suggested that, though we had optimized the mMRI sequence to aid in differentiation between the tumor and the surrounding bright subcutaneous fat tissue, mMRI-based manual segmentation by ITK and semiautomatic segmentation by 3D Slicer were significantly different from the true tumour volume, which indicated that mMRI-based segmentation was not ideal for GTV measurement. However, no significant differences were observed between DWI-based volume measurements and the true tumour volume when using either the manual or semiautomatic segmentation method. These findings suggest that the margins of tumorous tissue in DWI were obvious because of the high tumour cellularity, which was shown as restricted diffusion with higher signal intensity for DWI.

When comparing the performance of these two segmentation methods in our study, no significant difference was observed between manual (ITK) and semiautomatic (3D Slicer) segmentation when they were applied to mMRI or DWIs, which is consistent with other studies^22^,^24^.

Our results showed that a b value of 0 s/mm^2^ resulted in the best measurement reproducibility. This might be explained by the higher signal to noise ratio (SNR) with the lower b value, resulting in increased image quality and facilitating tumour volume definition when compared with higher b values^3^.

In addition, our results demonstrated better intra-observer agreement and GTV measurement reproducibility in larger tumours, which might be explained by the measurement error for a given measurement method being relatively smaller in larger tumours compared to smaller tumours. This hypothesis could also explain the interesting finding that irregularly shaped tumours demonstrated better intra-observer agreement and higher measurement reproducibility compared to regular tumours because irregular tumours are usually larger.

As presented by Wolf *et al.*^13^, the image quality for DWIs of subcutaneous xenografted tumours at the flanks was affected by image distortions due to strong susceptibility gradient artefacts at the air-tissue interface. Therefore, to address this problem, we wrapped the conscious mouse in sliced lean meat and fixed it with tape during image acquisition; we achieved satisfactory imaging quality with all DWI acquisitions, which contributed to more accurate segmentations in GTV measurements.

Our study provides several contributions. First, we compared different non-invasive GTV measurement methods with *ex vivo* specimen volume as the true tumour volume and revealed that DWI-based segmentation results in better accuracy of GTV measurements than both the mMRI-based segmentation and formula methods. Second, we compared the intraclass reproducibility of DWI-based semiautomatic segmentation (3D Slicer) with manual segmentation (ITK) and concluded that the semiautomatic segmentation method led to higher reliability. Therefore, DWI-based semiautomatic segmentation using 3D Slicer could be applied as a starting point for tumour volume measurements. Additionally, we observed that different b values showed excellent GTV measurement reproducibility in semiautomatic segmentation, especially when the b value was 0 s/mm^2^.

Our study has a couple of limitations. First, the mMRI sequence we used in our study is a modified T2WI rather than the conventional T1WI or T2WI sequence. Because the image contrast acquired with the modified sequence has signal composition from both T1WI and T2WI due to the short TR and long TE, it offers clear tumor boundary delineation from the bright subcutaneous fat tissue as well as from the isointensity wrapping lean meat used for stabilizing the animal. Another choice for morphological MRI is fat-suppressed T2WI sequence, which has similar contrast to DWI of b value of 0 s/mm^2^, may allow a fairer comparison between mMRI and DWI segmentation. However, the initial design of the mMRI protocol was just to optimize the tumor to surrounding tissue contrast, which was achieved with the currently used mMRI sequence parameters. The study results demonstrated that the GTV measured based on this mMRI sequence is significantly larger than true tumour volume and DWI-based measurements. This finding is contradictory to popular belief that mMRI, with its less distortion and better spatial resolution to DWI, should provide a more accurate GTV^13^. One possible reason could be the image contrast we acquired, therefore future studies comparing to the conventional T2WI sequence with or without fat suppression will be explored. Second, the time needed for semiautomatic and manual volume measurements of tumours was not recorded, although many studies have shown that semiautomatic segmentation can reduce the target delineation time in other tumour sites^25^,^26^,^27^. Third, we only assessed intra-observer agreement for all GTV measurements.

In conclusion, DWI-based semiautomatic segmentation with 3D Slicer allows for more accurate and reproducible GTV measurements of the subcutaneous tumour-xenografted nude mouse model. We suggest that semiautomated segmentation with 3D Slicer for DWI should be used to evaluate the response to anti-tumour therapies in animal models.

## Methods

### Animals and tumour model

The Animal Ethical Committee of Guangdong General Hospital approved this animal study. The methods were performed in accordance with relevant guidelines and regulations. The nude mice (BALB/c-nu) were provided by Guangdong Medical Laboratory Animal Center. Hepatocellular carcinoma cells (MHCC-97) were obtained by legal jurisdiction and were implanted subcutaneously into the right armpit of male nude mice (n = 35). Three to seven weeks after implantation, subcutaneous tumours with different sizes and shapes were measured *in vivo* by external callipers and MRI segmentation.

### Tumour measurement by the formula method with an external calliper

To determine the size of subcutaneous tumours, the greatest longitudinal diameter (A) and the greatest transverse diameter (B) were estimated with external callipers. Tumour volume was obtained from the modified ellipsoidal formula^5^: V = 1/2(AB^2^). All volume measurements were performed twice at an interval of 5 minutes by the same observer to generate intra-observer agreement.

### MR image acquisition

To avoid possible physiological changes, we did not use anaesthesia^28^. Therefore, we used sliced lean meat obtained in the supermarket to wrap around the conscious mouse that we fixed with tape before scanning the subcutaneous tumour, and we ensured that there was no gap between the meat and the tumour. Another advantage of this method was that it reduces magnetic susceptibility artefacts caused by the air-tissue interface. All MRI scans were performed with a receiver mouse coil (50-mm inner diameter, four-channel) in 1.5-T MRI scanners (Achieva, Philips Healthcare, The Netherlands). The examination protocol included a morphological MRI (mMRI) and DWI. In our study, the sequence for mMRI acquisition was optimized by modifying the conventional T2WI sequence (TR/TE = 571.2/80 ms, slice = 2.5 mm, gap = 0 mm, FOV = 50 × 50 × 28 mm^3^, matrix = 100 × 100). The axial DW MRI was acquired with a single-shot SE-EPI (spin-echo echo planar imaging) sequence (diffusion directions = 3; 12 b-values = 0, 10, 20, 30, 40, 50, 75, 100, 150, 300, 500, 800 s/mm^2^; TR/TE = 1464.2/81.6 ms; slice = 2.5 mm; gap = 0 mm; FOV = 60 × 60 × 12 mm^3^; matrix = 64 × 65) with fat suppression achieved by SPIR (spectral presaturation inversion recovery).

### Tumour surgical resection and ex vivo volume measurement

After MR scanning was completed and the integrity of the data was confirmed by a radiologist (Z.L.), each mouse was sacrificed, the tumour was completely resected and the specimen volume was measured twice using the water displacement method by one observer; the average volume from two measurements was considered the true tumour volume^11^. To reduce underestimation caused by tumour shrinkage, measurements were performed without fixation^10^. The specifications of the measuring cylinder we used was 10 ml or 25 ml depending on the size of the tumour.

### Image analysis

All MR images were downloaded from the MR scanner database onto a standalone workstation (Windows 8.1, Dell, USA) and then successively run on two types of open volumetric analysis software by a trained radiologist with 5 years of experience in MRI and DWI interpretation. Considering the heavy workload required to deal with DWI with 12 b values, we only chose 3 representative sets of DWIs with two smaller b values (0 s/mm^2^, 20 s/mm^2^) and one higher b value (800 s/mm^2^) for image segmentation.

For the evaluation of manual delineation, we used the freely available software ITK-SNAP (version 3.2, http://www.itksnap.org) to manually outline the visible tumour on each slice of the mMRI and 3 different DWI sets (b = 0, 20, 800 s/mm^2^). After tracing, a 3D reconstruction of the tumour was generated, and the total tumour volume was automatically calculated. Ambiguities in outlining the tumours in the axial plane were cross-checked by outlining the tumours in the corresponding sagittal and coronal planes^4^.

**Figure 1:**
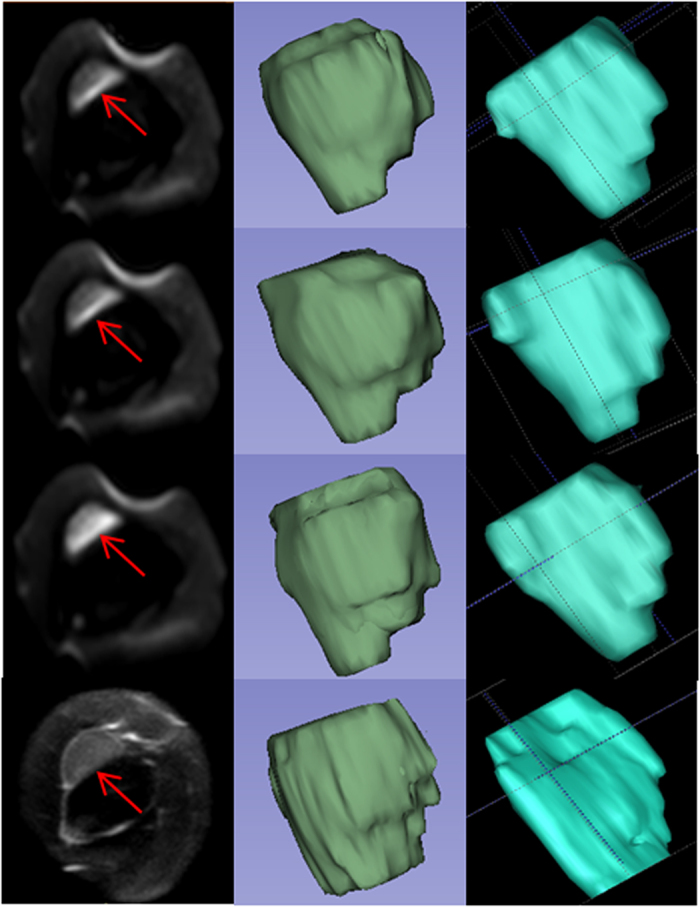
MRI-based tumour volume segmentations with the semiautomatic and manual segmentation methods. The leftmost images present the same subcutaneous tumour (red arrows) on an axial slice: b = 0 s/mm^2^ DWI (uppermost), b = 20 s/mm^2^ DWI (second image from the top), b = 800 s/mm^2^ DWI (third image from the top), and mMRI MRI (lowest). Moreover, comparison of total tumours in corresponding sequences obtained from semiautomatic segmentation by 3D Slicer (green, middle images) and manual segmentation by ITK (blue, rightmost images) are presented.

To evaluate semiautomatic delineation, tumour segmentation was performed with 3D Slicer software (version 4.3, http://www.slicer.org), which is an extensible algorithm and free platform for semiautomatic segmentation^29^. In the Slicer “Editor” module, we used the GrowCut algorithm followed by additional operations (such as painting, erosion and dilation), and then the semiautomatic segmentation results were output and inspected; in some cases, additional editing was required to achieve a satisfactory boundary^19^. In Fig. 1, DWIs (b = 0, 20, 80 s/mm^2^) and axial mMRI on the same slice are illustrated in the leftmost column. Moreover, 3D volumetric reconstructions of the subcutaneous tumour generated from 3D Slicer (green, middle images) and ITK (blue, rightmost images) were compared. Images were assessed in a random order, and the observer was blinded to the results produced by other methods.

### Statistical Analysis

Data are expressed as the mean ± standard deviation (SD). Normality was tested using the Kolmogorov-Smirnov method and then variance homogeneity was tested with the Levene test.

A paired Student’s t-test was performed to evaluate the systematic bias of repeated volume measurements by *in vivo* MRI and external calliper; if there was no significant difference between the two measurements, the average volume from the two measurements for all methods was compared with the true tumour volume obtained with the water displacement method.

To assess the effects of tumour size on GTV measurement, animals were divided into 2 groups based on the median size of the true volume (1.13 cm^3^ in this study). We also investigated the effects of tumour shape on GTV measurements by dividing tumours into 2 groups^6^: regular-shaped (round to ellipsoid) with smooth margins (n = 19) and irregular-shaped with multilobular margins (n = 16).

Results from all methods were compared with regard to tumour size and shape. Tumour volumes obtained by external callipers, and MRI-based segmentations were compared with the true tumour volume with the paired Student’s t-test for data of equal variance or Wilcoxon Rank Sum Test for data of unequal variance (see Supplementary Table S1 online).

Comparison between the manual and semiautomatic segmentation methods for mMRI or DWIs was also analysed with a paired Student’s t-test or Wilcoxon Rank Sum Test (see Supplementary Table S2 online).

If there was no significant difference between the true tumour volume and a given measurement method, the intraclass correlation coefficient (ICC) was used to assess the reproducibility of tumour measurement methods with external callipers or MRI-based segmentations.

Statistical analyses were performed with SPSS Statistics (version 22.0, IBM Corporation, America) and MedCalc software (version 15.2.2, http://www.medcalc.org). The tests were two-tailed, and a value of P < 0.05 was considered statistically significant. An ICC greater than 0.75 was regarded as being in good agreement^30^.

## Additional Information

**How to cite this article**: Ma, Z. *et al.* MR Diffusion-weighted imaging-based subcutaneous tumor volumetry in a xenografted nude mouse model using 3D slicer: a precise and repeatable method. *Sci. Rep.* **5**, 15653; doi: 10.1038/srep15653 (2015).

## Supplementary Material

Supplementary Information

## Figures and Tables

**Table 1:** Subcutaneous gross tumour volume (cm^3^) obtained by different methods.

Groups	Formula	ITK				3D Slicer				True TumourVolumes
		mMRI	DWI_b0	DWI_b20	DWI_b800	mMRI	DWI_b0	DWI_b20	DWI_b800	
General	1.99 ± 1.40	1.77 ± 1.36	1.72 ± 1.34	1.73 ± 1.33	1.73 ± 1.34	1.77 ± 1.35	1.73 ± 1.35	1.73 ± 1.35	1.73 ± 1.35	1.72 ± 1.35
<1.13 cm^3^	1.06 ± 0.21	0.82 ± 0.20	0.79 ± 0.18	0.80 ± 0.18	0.80 ± 0.17	0.82 ± 0.19	0.80 ± 0.18	0.80 ± 0.18	0.79 ± 0.18	0.79 ± 0.18
≥1.13 cm^3^	2.97 ± 1.45	2.78 ± 1.34	2.71 ± 1.34	2.71 ± 1.34	2.72 ± 1.34	2.77 ± 1.34	2.72 ± 1.35	2.72 ± 1.35	2.72 ± 1.34	2.71 ± 1.35
Regular	1.54 ± 0.90	1.39 ± 0.87	1.33 ± 0.83	1.35 ± 0.83	1.33 ± 0.82	1.37 ± 0.84	1.34 ± 0.83	1.34 ± 0.84	1.34 ± 0.84	1.33 ± 0.84
Irregular	2.51 ± 1.71	2.23 ± 1.70	2.19 ± 1.68	2.19 ± 1.67	2.20 ± 1.69	2.24 ± 1.70	2.20 ± 1.69	2.10 ± 1.69	2.20 ± 1.69	2.19 ± 1.69

Note: Data are presented as the mean ± standard deviation. The formula refers to the tumour volume calculated by V = 1/2(AB^2^), “A” refers to the greatest longitudinal diameter, “B” refers to the greatest transverse diameter. ITK and 3D Slicer refer to MRI-based segmentation using ITK and 3D Slicer software, respectively. “True Tumour Volumes” refer to specimen volumes measured by water displacement. General refers to all tumours. Size refers to all tumour volumes divided into two groups by the median of the true tumour volumes (1.13 cm^3^). Shape refers to all tumours divided into two groups based on their appearance. mMRI, morphological MRI; DWI_b0, diffusion-weighted imaging acquired with b value of 0 s/mm^2^; DWI_b20, diffusion-weighted imaging acquired with b value of 20 s/mm^2^; DWI_b800, diffusion-weighted imaging acquired with b value of 800 s/mm^2^.

**Table 2:** Comparison of gross tumour volumes obtained with the formula and MRI-based segmentation methods by ITK and 3D Slicer with the true tumour volume.

Group comparison	Size		Shape		General
	<1.13 cm^3^	≥1.13 cm^3^	Regular	Irregular	
Formula *VS* true tumour volume	0.000*	0.037*	0.000*	0.017^	0.000^
ITK *VS* true tumour volume					
mMRI	0.011^	0.000*	0.002^	0.002^	0.000^
DWI_b0	0.767	0.667	0.551	0.866	0.596
DWI_b20	0.050	0.787	0.070	0.661	0.060
DWI_b800	0.631	0.793	0.972	0.497	0.671
3D Slicer *VS* true tumour volume					
mMRI	0.001*	0.025^	0.003*	0.013^	0.000^
DWI_b0	0.171	0.504	0.278	0.506	0.092
DWI_b20	0.183	0.163	0.398	0.088	0.064
DWI_b800	0.586	0.403	0.608	0.163	0.351

Note: P values were obtained with the paired Student’s T test or the Wilcoxon Rank Sum test. *Significance at the 5% level was determined with a paired Student’s t-test; ^significant at the 5% level determined by the Wilcoxon Rank Sum test. ITK and 3D Slicer refer to MRI-based segmentation using ITK and 3D Slicer software, respectively. “True tumour volumes” refers to specimen volumes measured by water displacement. General refers to all tumours. Size refers to all tumour volumes divided into two groups by the median of the true tumour volumes (1.13 cm^3^). Shape refers to all tumours divided into two groups based on their appearances. mMRI, morphological MRI; DWI_b0, diffusion-weighted imaging acquired with b value of 0 s/mm^2^; DWI_b20, diffusion-weighted imaging acquired with b value of 20 s/mm^2^; DWI_b800, diffusion-weighted imaging acquired with b value of 800 s/mm^2^.

**Table 3:** Group comparison of gross tumour volumes between manual and semiautomatic segmentation based on MRI with regard to size and shape.

Group comparison	Size		Shape		General
	<1.13 cm^3^	≥1.13 cm^3^	Regular	Irregular	
ITK_mMRI vs3D-Slicer_mMRI	0.443	0.054	0.081	0.877	0.232
ITK_DWI_b0 vs3D-Slicer_ DWI_b0	0.244	0.223	0.220	0.257	0.087
ITK_ DWI_b20 vs3D-Slicer_ DWI_b20	0.511	0.740	0.308	0.326	0.787
ITK_ DWI_b800 vs3D-Slicer_ DWI_b800	0.449	0.680	0.721	0.352	0.608

Note: P values were obtained from the paired Student’s T test or the Wilcoxon Rank Sum test. No significant difference was observed between these two segmentation methods. ITK and 3D-slicer refer to MRI-based segmentation using ITK and 3D-slicer software, respectively. General refers to all tumours. Size refers to all tumour volumes divided into two groups by the median of the true tumour volumes (1.13 cm^3^). Shape refers to all tumours divided into two groups based on their appearance. mMRI, morphological MRI; DWI_b0, diffusion-weighted imaging acquired with b value of 0 s/mm^2^; DWI_b20, diffusion-weighted imaging acquired with b value of 20 s/mm^2^; DWI_b800, diffusion-weighted imaging acquired with b value of 800 s/mm^2^.

**Table 4:** Intra-observer agreements for gross tumour volume measurements with different segmentation methods based on DWIs.

Groups	Size		Shape		General (n = 35)
	<1.13 cm^3^ (n = 18)	≥1.13 cm^3^(n = 17)	Regular (n = 19)	Irregular (n = 16)	
ITK					
DWI_b0	0.9920(0.9789,0.9970)	0.9998(0.9995,0.9999)	0.9995(0.9988,0.9998)	0.9999(0.9997,1.0000)	0.9998(0.9997,0.9999)
DWI_b20	0.9908(0.9755,0.9965)	0.9997(0.9993,0.9999)	0.9994(0.9984,0.9998)	0.9999(0.9997,1.0000)	0.9996(0.9992,0.9998)
DWI_b800	0.9908(0.9755,0.9965)	0.9993(0.9981,0.9997)	0.9996(0.9991,0.9999)	0.9996(0.9989,0.9999)	0.9997(0.9993,0.9998)
3D Slicer					
DWI_b0	0.9978(0.9942,0.9992)	0.9999(0.9996,1.0000)	0.9998(0.9995,0.9999)	0.9999(0.9998,1.0000)	0.9999(0.9998,1.0000)
DWI_b20	0.9965(0.9908,0.9987)	0.9999(0.9997,1.0000)	0.9998(0.9995,0.9999)	0.9999(0.9998,1.0000)	0.9999(0.9998,1.0000)
DWI_b800	0.9959(0.9890,0.9985)	0.9999(0.9997,1.0000)	0.9998(0.9994,0.9999)	0.9999(0.9999,1.0000)	0.9999(0.9998,1.0000)

Note: Data are represented as intraclass coefficients and the number in parentheses are 95% confidence intervals. ITK and 3D Slicer refer to MRI-based segmentation using ITK and 3D Slicer software, respectively. General refers to all tumours (n = 35). Size refers to all tumour volumes divided into two groups by the median of the true tumour volumes (1.13 cm^3^). Shape refers to all tumours divided into two groups based on their appearance. DWI_b0, diffusion-weighted imaging acquired with b value of 0 s/mm^2^; DWI_b20, diffusion-weighted imaging acquired with b value of 20 s/mm^2^; DWI_b800, diffusion-weighted imaging acquired with b value of 800 s/mm^2^.
