# Supplementary material for: MR diffusion-weighted imaging-based subcutaneous tumour volumetry in a xenografted nude mouse model using 3D Slicer: an accurate and repeatable method
Source: Sci Rep. 2015 Oct 22;5:15653. doi: 10.1038/srep15653 (PMC4614907; doi:10.1038/srep15653)
Supplement: Supplementary Information [file srep15653-s1.pdf]

MR diffusion-weighted imaging-based subcutaneous tumour volumetry in a xenografted nude mouse model using 3D Slicer: an accurate and repeatable method

Zelan Ma[1,2+], Xin Chen[3+], Yanqi Huang[1,2], Lan He[1,4], Cuishan Liang[1,2], Changhong Liang[1*], Zaiyi Liu[1*]

[1]Department of Radiology, Guangdong General Hospital, Guangdong Academy of Medical Sciences, Guangzhou, Guangdong, 510080, China,

[2]Graduate College, Southern Medical University, Guangzhou, Guangdong, 510515, China,

[3]Department of Radiology, The Affiliated Guangzhou First Hospital, Guangzhou Medical University, Guangzhou, Guangdong, 510180, China,

[4]School of Medicine, South China University of Technology, Guangzhou, Guangdong, 510006, China.

Corresponding author:

Zaiyi Liu (zyliu@163.com); Tel: +86 20 83870125

106 Zhong Shan Er Lu, Guangzhou, Guangdong Province 510080, China

OR

Changhong Liang (cjr.lchh@vip.163.cm); Tel: +86 20 83870125

106 Zhong Shan Er Lu, Guangzhou, Guangdong Province 510080, China

Other authors' email addresses are as follows: Z.M. (zelanma@163.com); X.C. (wolfchenxin@sina.com); Y.H. (54152412@qq.com); L.H. (925842821@qq.com); C.L. (405821317@qq.com).

[+]These authors contributed equally to this work.

Supplemental Table 1. Statistical methods used for between-group comparison of gross tumour volumes obtained with the formula and MRI-based segmentation methods by ITK and 3D Slicer with the true tumour volume

Group comparison	Size		Shape		General
	<1.13 cm^3	⩾1.13 cm^3	Regular	Irregular	
Formula *VS* true tumour volume	T test	T test	T test	Wilcoxon test	Wilcoxon test
ITK *VS* true tumour volume					
mMRI	Wilcoxon test	T test	Wilcoxon test	Wilcoxon test	Wilcoxon test
DWI_b0	T test	T test	T test	T test	T test
DWI_b20	T test	T test	T test	T test	Wilcoxon test
DWI_b800	T test	T test	T test	T test	T test
3D Slicer *VS* true tumour volume					
mMRI	T test	Wilcoxon test	T test	Wilcoxon test	Wilcoxon test
DWI_b0	T test	T test	T test	T test	Wilcoxon test
DWI_b20	T test	Wilcoxon test	Wilcoxon test	Wilcoxon test	Wilcoxon test
DWI_b800	T test	T test	T test	Wilcoxon test	Wilcoxon test

Note: T test refers to Student's T test; Wilcoxon test refers to Wilcoxon Rank Sum test. ITK and 3D Slicer refer to MRI-based segmentation using ITK and 3D Slicer software, respectively. "True tumour volumes" refers to specimen volumes measured by water displacement. General refers to all tumours. Size refers to all tumour volumes divided into two groups by the median of the true tumour volumes (1.13 cm^3). Shape refers to all tumours divided into two groups based on their appearances. mMRI, morphological MRI; DWI_b0, diffusion-weighted imaging acquired with b value of 0 s/mm^2; DWI_b20, diffusion-weighted imaging acquired with b value of 20 s/mm^2; DWI_b800, diffusion-weighted imaging acquired with b value of 800 s/mm^2.

Supplemental Table 2. Statistical methods used for between-group comparison of gross tumour volumes between manual and semiautomatic segmentation based on MRI with regard to size and shape

Group comparison	Size		Shape		General
	<1.13 cm^3	⩾1.13 cm^3	Regular	Irregular	
ITK_mMRI vs 3D-Slicer_mMRI	T test	Wilcoxon test	T test	Wilcoxon test	Wilcoxon test
ITK_DWI_b0 vs 3D-Slicer_ DWI_b0	T test	T test	T test	T test	T test
ITK_ DWI_b20 vs 3D-Slicer_ DWI_b20	T test	Wilcoxon test	T test	Wilcoxon test	Wilcoxon test
ITK_ DWI_b800 vs 3D-Slicer_ DWI_b800	Wilcoxon test	T test	T test	Wilcoxon test	Wilcoxon test

Note: T test refers to Student's T test; Wilcoxon test refers to Wilcoxon Rank Sum test. ITK and 3D-slicer refer to MRI-based segmentation using ITK and 3D Slicer software, respectively. General refers to all tumours. Size refers to all tumour volumes divided into two groups by the median of the true tumour volumes (1.13 cm^3). Shape refers to all tumours divided into two groups based on their appearance. mMRI, morphological MRI; DWI_b0, diffusion-weighted imaging acquired with b value of 0 s/mm^2; DWI_b20, diffusion-weighted imaging acquired with b value of 20 s/mm^2; DWI_b800, diffusion-weighted imaging acquired with b value of 800 s/mm^2.
